# Effects of a Multi-Suckling System Combined With Enriched Housing Post-Weaning on Response and Cognitive Resilience to Isolation

**DOI:** 10.3389/fvets.2022.868149

**Published:** 2022-04-11

**Authors:** Severine P. Parois, Lisette E. Van Der Zande, Egbert F. Knol, Bas Kemp, T. Bas Rodenburg, J. Elizabeth Bolhuis

**Affiliations:** ^1^Adaptation Physiology Group, Wageningen University & Research, Wageningen, Netherlands; ^2^Epidemiology Health and Welfare Research Unit, Ploufragan-Plouzané-Niort Laboratory, French Agency for Food, Environmental and Occupational Health and Safety (ANSES), Ploufragan, France; ^3^Topigs Norsvin Research Center, Beuningen, Netherlands; ^4^Animals in Science and Society, Faculty of Veterinary Medicine, Utrecht University, Utrecht, Netherlands

**Keywords:** *Sus scrofa*, challenge, enrichment, recovery, alternative, cognition

## Abstract

Improving welfare is still a critical issue in pig husbandry. Upgrades of the housing environment seem to be a promising solution to optimise resilience as a whole, and therefore improve animal welfare. The objective of this study was to evaluate the effect of an alternative housing system to enhance cognitive resilience and also to promote the pigs' welfare. A total of 96 piglets from two contrasted housing systems [alternative housing system (AHS) vs. conventional system (CONV)] was used. The major upgrades of the alternative system were multi-litter housing during lactation, delayed weaning, extra space allowance, and environmental enrichment from birth onwards. To estimate welfare, weight, and feed intake (as a general indicator of performances), the tear staining area (as a chronic stress indicator), behavioural postures, heart rate traits, and saliva cortisol concentration were measured over a 21 h-isolation. To assess cognitive resilience, the pigs were subjected to a maze with a social reward both before and after the isolation challenge and indicators of cognitive abilities were followed. The AHS pigs showed lower cortisol levels and tear staining area before the challenge, demonstrating overall better welfare due to the alternative housing conditions. During the challenge, AHS pigs had a lower heart rate, higher heart rate variability, and higher vagal activity than the CONV pigs, which might indicate a reduced sensitivity to the stressor. AHS pigs appeared to have a better long-term memory tested in a maze. Providing social and environmental enrichments, that fit the satisfaction of the essential needs of the pigs better, appears to be beneficial for pig welfare as a whole. Its effects on cognitive resilience still need to be proven.

## Introduction

In many parts of Europe, but also in other parts of the world, pig production is no longer evaluated in terms of productivity alone (1). The public debate also focuses on the welfare of the animals, public health consequences, or the acceptability of animal management practises. Both from an economic and societal perspective, there is, therefore, an urgent need to reduce health and welfare problems in pigs. Throughout their life, animals experience several stressors that vary in terms of source and intensity. The ability to cope with stressors is strongly related to the resilience of animals. Resilience can be defined as: “the capacity of the animal to be minimally affected by a disturbance or to rapidly return to the physiological, behavioural, cognitive, health, affective, and production states that pertained before exposure to a disturbance” (2). This capacity to cope with perturbations and to restore homeostasis is important for the performance and welfare of farm animals, as well as for economic results (3–6). Optimising resilience is seen as a potential strategy to enhance welfare (2). Indeed, animals in a state of poor resilience may only need a small disturbance to collapse into a health or welfare crisis. Poor resilience may therefore manifest itself as an increased risk to develop behavioural and health problems (7). Increasing resilience in pig production systems is therefore not only a scientific challenge, it equally aims to respond to public concerns with regard to livestock farming and especially with regard to welfare concerns in pig production.

In spite of efforts made to improve pig health and welfare in existing systems (e.g., through legislation directing minimum husbandry standards), the health problems are currently still predominantly treated by antibiotics and other therapeutic drugs, and the behavioural problems are battled by mutilating animals (e.g., tail docking in pigs to prevent tail biting). The satisfaction of the essential behavioural needs of the animals, e.g., species-specific behaviours, with appropriate housing conditions seems to be a promising solution to improve their resilience (2, 8–10). A housing system that provides pigs with social and environmental enrichment more closely resembles the natural situation of feral and wild pigs (11–14). Environmental enrichment is defined as a modification in the environment that improves the biological functioning of animals (15). Enrichment in pig husbandry is typically applied to increase the opportunities to exhibit important natural behaviours, such as mother-young interactions (16) and exploration (17). Evidence is accumulating that pigs reared and kept under conditions that better meet their behavioural needs show less injurious behaviours (18–20), improved cognitive performance (21–23), and a more optimistic mood (24). Although favourable effects of such housing conditions on pig behaviour are widely reported, information about their impact on resilience is scarce. Recent studies indicate, however, that pigs housed in enriched environments are more resilient to disease, sickness, and transport challenges, as shown in a faster recovery and/or lower signs of accumulated stress (25, 26).

Intensively farmed pigs have to cope with the metabolic demands of rapid and efficient growth, combined with the multiple acute and chronic social and physical stressors they are exposed to. They suffer from these stressors in many ways, with symptoms ranging from easy startling to systemic inflammation, causing impaired welfare. The cumulative stress causes wear and tear on the animals and reduce their future capacity to recover from challenges. Exposure to stressors may also cause attention shifts, narrow attention, and decrease decision speed, and all of these factors influence the cognitive performance of pigs (27–29). Cognitive resilience can be described as the ability to overcome the negative effects of stressors on cognitive functions (30). Amongst the situations that have deleterious impacts, isolation is especially stressful for pigs, as they are social animals. Isolation has strong effects on stress indicators and can cause diverse physiological changes, like an increase in plasma cortisol concentrations, a decrease in body temperature, and in tumour necrosis factor-alpha (31–33). The repercussion of isolation stress on cognitive performance, in particular spatial memory, is unclear in the literature. Mendl et al. (27) reported that isolation stress negatively impacts spatial memory; while Van der Staay et al. (34) did not find any impact of overnight isolation on such memory. Depending on the resilience status of animals, either a mild stressor (e.g., a few hours of isolation) for non-resilient individuals or a more severe stressor for resilient individuals may disrupt their cognitive functioning and result in inappropriate responses with negative repercussions for the individual or its pen mates. Housing conditions may influence the development of cognitive functions and modulate the general resilience of the individuals and their welfare (26, 35). Therefore, providing social and environmental enrichments is expected to have beneficial effects on both social isolation stress and cognitive resilience, although this has not been investigated yet. Increasing resilience may help animals to deal better with a stressor, thereby protecting animals from the negative consequences of this stressor for cognitive functioning, but also improving cognitive performance and cognitive resilience. An increased understanding of how these enriched housing conditions may influence cognitive resilience can lead to improvements in the welfare of pigs (36, 37).

This study aimed to investigate the effect of an alternative housing system, consisting of multi-litter housing during lactation, delayed weaning, and extra space allowance and environmental enrichment at all times, as compared with conventional commercial conditions on the response of piglets to social isolation stress and their cognitive resilience after this isolation challenge.

## Materials and Methods

Established principles of laboratory animal use and care and the Dutch law on animal experiments were followed. They comply with the European Directive 2010/63/EU on the protection of animals used for scientific purposes. The Animal Care and Use Committee of Wageningen University approved the experiment (AVD1040020186245). The sample size estimation was based on the rise in cortisol levels using results obtained in pigs subjected to isolation stress (31, 38, 39) (α = 5%, power = 80%, SD = 2.42, δ = 2.8).

### Animals

A total of 144 Tempo × Topigs-20 pigs (*n* = 71 females; *n* = 73 males) were used during the experiment, spread over three batches (*n* = 48 pigs per batch). The piglets were offsprings from 24 multiparous sows. During lactation, half of the sows and their piglets were housed in a conventional farrowing pen (CONV; mean ± SD; sow parity = 4.2 ± 1.8) and the other half in an alternative group housing system (AHS; parity = 4.0 ± 1.7) at the Swine Innovation Centre (Sterksel, The Netherlands), see below for details. The piglets were not castrated, nor were their tails docked or teeth clipped. The average birth weight was similar for piglets from both systems: 1.46 ± 0.28 kg for the CONV and 1.44 ± 0.27 kg for the AHS.

### Housing Systems

#### From Birth to 9 Weeks of Age

The piglets were raised in two different housing systems [similar to van Nieuwamerongen et al. (20)]. The AHS was comprised of five farrowing pens of 3.2 × 2.2 m (mix of solid and slatted floor), adjacent to a communal area of 11.1 × 2.80 m (solid floor). Next to the communal area, a dunging area (2.8 × 3.3 m, slatted floor) and feeding area (4.2 × 3.3 m, solid floor) were situated. Enrichment was provided in the form of four jute bags and after birth, a slide of straw was added to the farrowing pens. One week before the expected farrowing date, five sows per batch were put in this system. Two days before the expected day of farrowing, sows were moved to a farrowing pen and confined in a farrowing crate. Two days after farrowing, they were allowed to access the full system again. Newly born piglets were kept per litter in the farrowing pens for 1 week. The piglets were provided with a heated piglet nest next to the farrowing pens (0.7 × 1.6 m), with a temperature of 33–35°C (day 1 till day 7), 29–31°C (day 7 till day 25), and 23–26°C (day 25 till weaning). After the 1st week of life, the piglets could access the entire system and mingle with the other litters. The piglets were fed in round bowls (until 5 weeks of age) and from a sensor-controlled automatic feeder (Rondomat, from 3 weeks of age). Besides this, the piglets could participate in feeding with the sows, which were fed in a large trough placed on the floor. Ingestion of solid feed was stimulated with the use of intermittent suckling from week 5 of age onwards (40). AHS piglets were weaned at an average of 62.6 ± 1.9 days and at a bodyweight of 26.6 ± 4.9 kg. They received a starter diet from 35 days onwards.

In the CONV system, the piglets were kept in farrowing pens of 2.8 × 1.8 m until weaning. Sows were confined in a crate. The floor consisted of metal slats within the crate. There was a solid floor of 1.2 × 0.3 m with a heating lamp for the piglets and the remaining area consisted of plastic slats. The piglets received additional creep feed in the farrowing pens from 1 week after birth. CONV piglets were weaned at 27.4 ± 1.2 days of age and 8.7 ± 1.3 kg. After weaning, CONV piglets were housed with their littermates in nursery pens of 3.18 × 1.0 m (0.40 m^2^ per piglet) for 5 additional weeks with a chain and jute bag as enrichment. They received a commercial weaner diet for 10 days after weaning and a starter diet, similar to that provided to AHS piglets, from 35 days onwards.

Lights were on from 07:00 till 19:00 in both systems, giving the sows and piglets a 12 h light regime with 115 Lux. Besides that, the AHS had natural daylight. The transition between day and night light settings was done progressively in 10 min. The ambient temperature was 23°C in both systems. Water was available *ad libitum* in both systems.

#### From 9 Weeks of Age Onwards

After the weaning of the AHS piglets, all piglets were moved to the Carus research facilities in Wageningen, the Netherlands, where they were mixed in groups of six unfamiliar piglets originating from the same system. Litter, sex, and weight were balanced between pens. Piglets were selected based on their sex (50:50% male and female), and weight at birth to have piglets representative of the full litter. Per litter, six piglets were selected: two piglets with a birth weight between the minimum weight of the litter +10% and the 1st weight quartile (light); two piglets with a birth weight between the 1st and 3rd quartile (medium); two piglets with a birth weight between the 3rd quartile and the maximum weight −10% (heavy). Four animals per pen (Focals, two males and two females) were exposed to the experimental challenges (see below), while two other pigs served as companions (the two extreme pigs deviating from the average pen weight most).

The CONV pigs were housed in standard pens of 1.20 × 4.67 m with conventional space allowance (0.93 m^2^ per pig), with a solid and slatted floor without substrate. The AHS pigs were housed in a 2.40 × 4.67 m pen, i.e., double the size of a conventional pen (1.87 m^2^ per pig), enriched with deep straw, peat, and sawdust bedding, which was replenished regularly (2.5 kg of straw and 30 L of sawdust every day, 22.5 L peat every week). Besides that, AHS pigs were provided with hay, egg trays, or alfalfa once a week and a chain, jute bag, or rope (rotation every week), plus one extra toy (either a biting ball on a chain, a free chewing ball for dogs, a tyre dog toy, a porcichew^®^ toy, a green MS Schippers Bite cylinder^®^, or a green MS Schippers Cross^®^) which was changed every 2 days. The CONV piglets were provided with a ball and a chain with screws, which were not changed. The AHS and CONV pens were placed alternately in the rooms. The pigs were all fed the same feed (a standard commercial diet for growing pigs) *ad libitum* from a single pig feeder and water was available *ad libitum*.

The light regime was similar to that before 9 weeks of age, giving the pigs 115 Lux in the pens during the day (from 7 to 19 h; 5,000 K ultraviolet A at an intensity of 42, 2,700 K at 60) and 30 Lux during the night (5,000 K ultraviolet A at an intensity of 3, 2,700 K at 0). The transition between the day and night rhythm was done progressively for 10 min. No natural daylight was available. The temperature was kept at 23°C for the first 2 days, then at 22°C for the 2 subsequent days and at 21°C onwards.

### Isolation Challenge

At the age of 76.4 ± 1.4 days (weight: 35.7 ± 4.9 kg), the 96 focal pigs were isolated for 21 h in a separate room. Due to time and space constraints, the pigs were isolated on 3 consecutive days, balanced across treatments. The isolation pens were similar for every single pig with a dimension of 1.2 × 2.85 m. The pens were made with solid walls to prevent any physical or visual contact between the pigs kept in the same room. They contained an individual feeder and a drinker. Enrichment consisted of one chain with screws and one chain with a ball for every pig, plus an extra jute sack for AHS pigs only to reduce the contrast with enrichment in the home pen. No bedding material was provided. The light schedule and temperature in the isolation pens were the same as in the home pen. The isolation challenge was video recorded using a camera from the ceiling (CCD colour camera, 480 lines, 1 lux/f2.0 320 kpix, 200 mA, Velleman, Belgium). A total of five saliva samples were collected to follow the response and recovery of salivary cortisol of the animals: 15 min before the isolation challenge (baseline), and 1, 3, 5, and 21 h after the beginning of the isolation. Pigs were allowed to chew for 1–2 min on the swabs which were held by clamp forceps. Pigs were habituated to the procedure previously.

The pigs were equipped with an accelerometer on the left rear leg (Pendant G Acceleration Data Logger, Onset Computer Corporation, Pocasset, MA, USA) to determine their postures; and with a heart rate belt (Zephyr BioHarness 3^™^). The equipment stayed on the pigs for 5 h after the entrance to isolation. The accelerometer on the leg was put on the outside of the leg such that the x-axis was parallel to the leg of the pig and pointing down. The tri-axial accelerometers were programmed to log at 1-s intervals on 3 axes. They had a measurement range of ±3 *g*, an accuracy of ±0.105 *g*, and a memory of 21.8 kB for combined *x*-, *y*-, and *z*-axis readings. For programming and reading out the accelerometers, a coupler, an optical base station with a USB interface, and the HOBOware Pro computer program (Onset Computer Corporation, Pocasset, MA, USA) were used. The data were transformed from g to postures with Microsoft Excel (Microsoft Corporation, Redmond, WA, USA). Postures were determined according to Ringgenberg et al. (41). The following algorithm was used to automatically compute the posture of the pigs: IF (X ≥ 130°) THEN posture = standing ELSE posture = lying. Postures maintained for <20 s were considered as artefact and corrected by the behaviour done by the pig just before. The percentage of time spent in each posture was determined over 15-min intervals.

Regarding the heart rate measurement, five sections of 10 min, one per hour, were extracted from the dataset from each pig for time and frequency domain analysis, together with non-linear (including geometric) analysis. The 10 min sections were selected within the first 20 min of each hour paying attention to the quality of the data. The Kubios artefact correction philtre (threshold: very strong) was used and only sections with <25% artefacts corrections were analysed. The Kubios HRV Standard 3.3.1. software (Kubios Oy, University of Eastern Finland, Finland) (42) was used to obtain the heart rate variability (HRV) variables. The following three time-domain variables were examined: (1) interbeat interval (IBI) mean (or risk ratio (RR) mean; RR is the interval between successive R peaks of the QRS complex of the Electrocardiogram wave). The RR mean provides information on the heart rate (43); (2) root mean square of successive RR differences (RMSSD), which reflects the integrity of vagus nerve-mediated autonomic control of the heart; and (3) the SD of all RR intervals of the dataset (SDNN), which is a good predictor of overall variability present at the time of recording. Frequency domain analysis was done using a Fast Fourier Transformation (FFT) obtaining high (HF), and low frequency (LF) bands, expressed in normalised units (n.u.). Frequency bands widths (LF: 0.01–0.09 Hz; HF: 0.09–2.0 Hz) were assigned according to pig recommended ranges (44). The following two frequency domain variables were examined: (1) LF:HF ratio, also referred to as the Sympathetic Nervous System indicator (SNSI), which reflects sympathetic activity, and (2) HF/total power and pNN50, the Parasympathetic Nervous System indicator (PNSI), used to enumerate vagal activity (43). For geometric analysis, a Poincaré plot was plotted in Kubios and SD1 (short-term variability) and SD2 (long-term variability) was calculated. The following geometric variable was examined: (1) SD1:SD2 ratio, which is an indicator of sympathetic tone. The software Kubios also estimated Baevsky's stress index, which is a geometric measure of heart rate variability indicative of both sympathetic activity and central regulation [see Sahoo et al. (45) for more details about the calculation].

**Figure 1:**
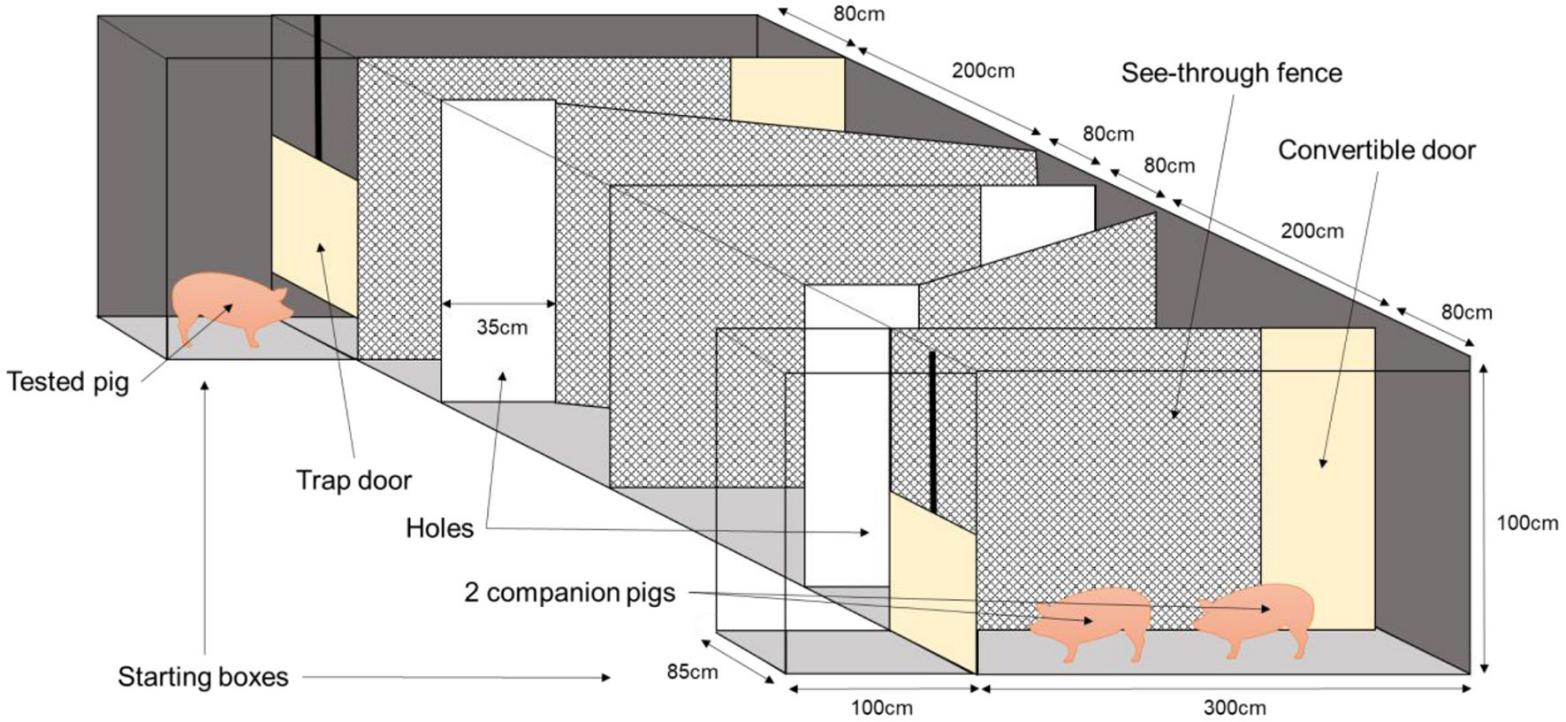
Schematic view of the maze used for the cognitive test, with the tested pig starting on the opposite side of the two companion pigs.

Immediately before and after being isolated, the pigs were subjected to a cognitive test to estimate the impact of the isolation challenge on their cognitive resilience. The pigs had to walk 10 m in a short corridor to move from the isolation pen to the maze and vice versa, which took less than a minute. The pigs' working memory was tested with a maze based on social motivation. The companion pigs acted as social support and “reward” at the end of the maze. The arena (4 × 7.30 m) was located in a sound-attenuated test room and consisted of dark hardboard walls with a height of 1 m and a grey concrete floor. The ambient temperature in the room of the maze was 21°C, the same as that in the home pen and isolation pen, and the light intensity was 110 Lux. The focal pig started at one side of the maze, while two companion pigs from its pen were at the other end. All the walls were made of a see-through fence, so the pig could see the companion pigs, besides smelling and hearing them. The walls were skewed, to make the maze more complex (Figure 1). The pig started in a starting box with a guillotine door on either one of the two sides of the maze. The side of the maze was used as starting point before isolation was balanced for housing. Time started as soon as the two front paws of the pig had left the starting box. To reach the companions, the pig had to find the openings at the end of the maze's corridors. Each pig was subjected to four consecutive trials before and four consecutive trials after social isolation. A trial could end in two different ways: when the pig had fully crossed (all four legs) the fourth opening; or when the trial reached the maximum time allowed to solve the task (5 min for the first trial, 3 min for the second and 1.5 min for trials 3 and 4). At the end of the trial, the tested pig was gently pushed out of the arena and brought back to the start box or back to its pen if it was its last trial. After being isolated, the pigs were tested again to see if they would perform differently after the social challenge, to get an indication of cognitive resilience. After social isolation pigs started at the other side of the maze than before isolation. This was done to secure the testing of short-term working memory, rather than the use of the long-term memory and to avoid a possible laterality effect. Behaviours (move, stand, lie, sit, push the fence, defecate, and urinate) were continuously scored by a trained observer during the test using a Psion hand-held computer with the Observer 5.0 software package (Noldus Information Technology, Wageningen, The Netherlands). The behaviours “lie,” “sit,” “defecate,” and “urinate” barely happened and were therefore not statistically analysed. A second trained observer recorded the latencies for the tested pig to cross each hole with its 2 front legs starting from the beginning of the test and to finish the trials. As a measure of working memory, the improvement in the pig's duration to reach each hole within days as well as between days and the percentage of time pushing the fence over the four trials were assessed.

### Indicators Measured During the Challenges

The pigs were weighed 24 h before and 24 h after the challenge. Relative weight gain was estimated as follows: $\frac{\left(Final\;weight\;-Initial\;weight\right)}{Initial\;weight}$. The feed intake over the 21 h-period was also measured. Photographs of the left eye of each focal pig were taken right before and 24 h after isolation. Measurements of tear staining were made on photographs by a single experienced person, blind to treatment, using the ImageJ software (National Institute of Health, Bethesda, USA) (46) to delimit the tear perimeter. The length of the iris was used as a scale to standardise the measurements. All the brownish areas on the direct periphery of the eye (bottom of the upper eyelid, top of the lower eyelid, internal and external corners) were recorded (33). The variable analysed was the cumulative area covered by the stain.

### Saliva Samples

Saliva was collected with Salivettes^®^ containing polypropylene swabs (Sarstedt Inc 51.1534.500) for cortisol determination. The Salivettes^®^ were centrifuged at 1,500 *g* for 10 min at room temperature. Saliva was stored at −20°C until laboratory analyses. Cortisol assays were performed using the cortisol ELISA kit from IBL (ref RE52611, Germany).

### Statistical Analyses

Statistical analyses were performed with the software R 4.0.3. (R Foundation for Statistical Computing, Austria) (47). The variables' latencies to cross the holes in the maze, tear staining, and saliva cortisol concentration were normalised by logarithmic transformation. Residuals of other variables were normal without transformation. Areas under the recovery curves were approximated from repeated measurements using the trapezoidal rule $\overset{N}{\underset{k=0}{\sum }}\left(\frac{f\left(x_{k-1}\right)+f\left(x_{k}\right)}{2}\right)\times \Delta xk$, where *f(x)* is a function and Δ*x*_*k*_ is the length of the *k*-th subinterval.

On focal pigs only, linear mixed models with the function lmer from the R package “lme4,” were used for all variables measured once. In these models, housing and sex (boar vs. gilt) were fixed effects, and batch and pen were random effects. Repeated variables were analysed with a linear mixed model with housing, sex, time, and the interaction housing × time as fixed effects and the pen, batch, and pig identification as random effects. For the heart rate variables, the percentage of the artefact was added to the model as a fixed effect. For the variables measured in the maze, the time effect was substituted by the day, the trial, and their interaction day × trial which involved the inclusion of the other interactions housing × day, housing × trial and housing × day × trial as well, and the random effect pig identification within the day was added.

*P*-values below 0.05 were considered as significant effects and below 0.1 as tendencies. When a significant effect was found, pairwise comparisons between groups were made with the emmeans function of the emmeans package from R, including a Tukey correction.

## Results

### Response to the Isolation Challenge

Relative weight gain (1.07 ± 0.17 %) and feed intake (1.24 ± 0.03 kg) were not affected by housing or sex.

Tear staining was reduced after the challenge (time effect *p* < 0.0001: 0 h = 0.30 ± 0.069, 21 h = 0.19 ± 0.069) and CONV pigs had a larger area (0.31 ± 0.071) than AHS pigs (0.18 ± 0.071, *p* = 0.0006). The interaction housing × time tended to be significant (*p* = 0.090) with CONV pigs presenting higher values before the challenge than after (0.39 ± 0.073 and 0.24 ± 0.073, respectively), whereas tear staining areas were similar before (0.21 ± 0.073) and after (0.15 ± 0.073) the challenge for the AHS pigs.

**Figure 2:**
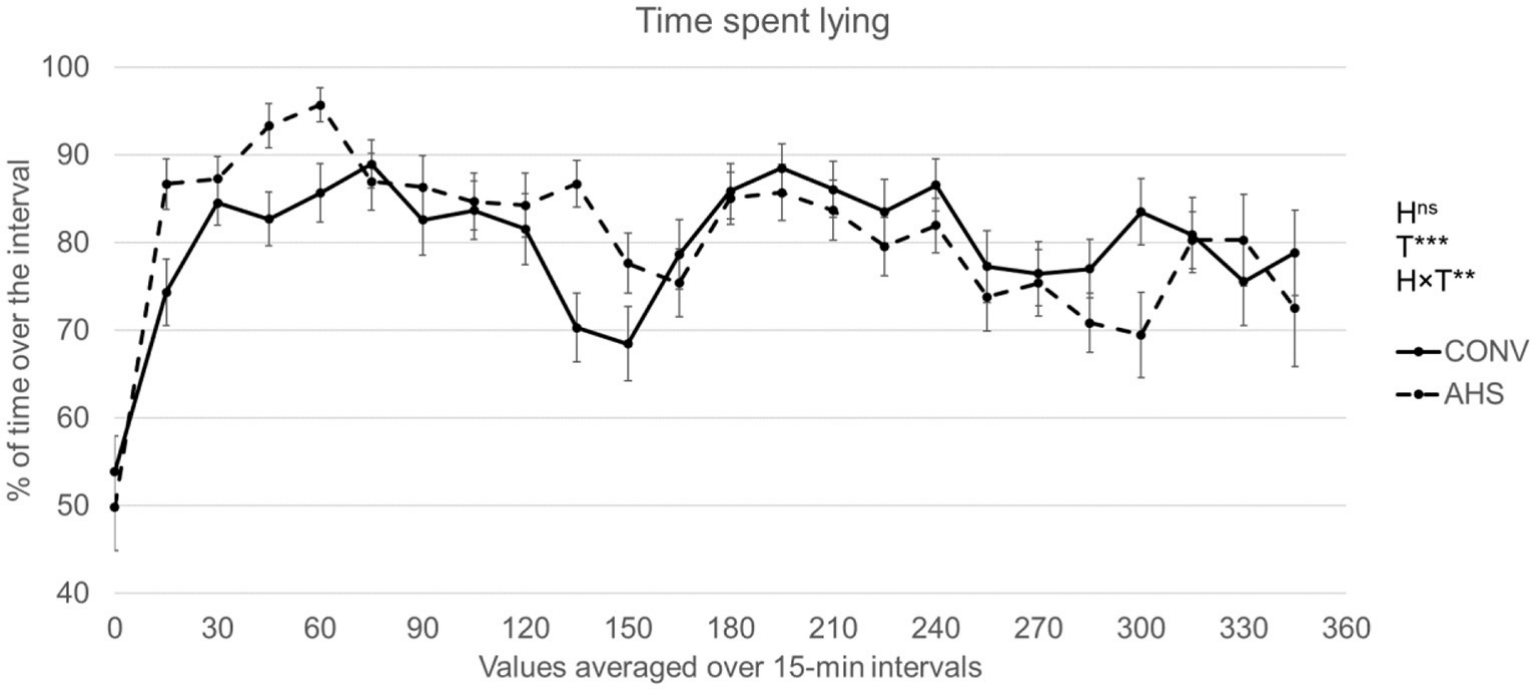
Time spent lying of pigs housed in an alternative (AHS) or conventional system (CONV) measured during the first 6 h of an isolation challenge with an accelerometer. H, housing effect; T, time effect; H × T, housing × time interaction; ***P < 0.001, **P < 0.01, ^ns^not significant.

The time spent lying over the first 6 h of isolation (Figure 2) was affected by time (*p* < 0.0001) and by the interaction housing × time (*p* = 0.0032). For both groups, during the first quarter of isolation, the pigs spent <50% of their time lying, while it was about 80% of the time for the rest of the period. There was no housing effect within the time period. However, for the CONV pigs, at 150 min of isolation, there was a significant drop (68.5% of time spent lying as compared with the averaged 80.9% observed on the other intervals). For the AHS pigs, between 45 and 75 min, there was a significant increase in lying duration (94.5% as compared with the 80.7% for the rest of the period).

**Figure 3:**
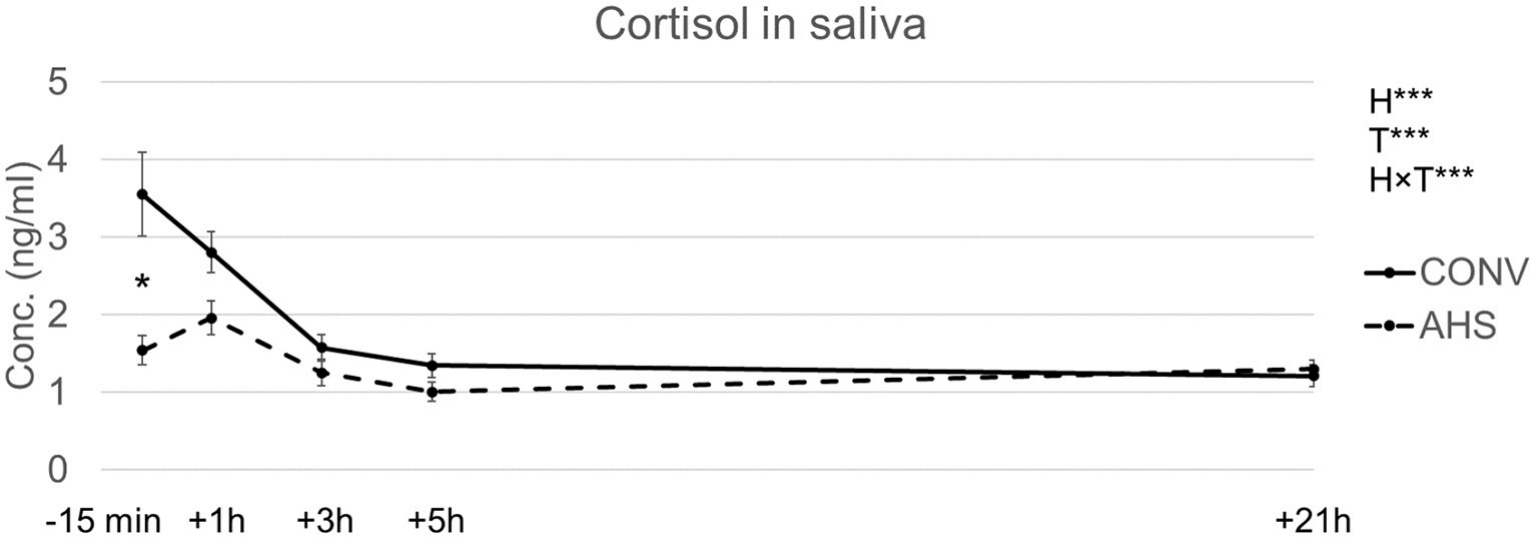
Saliva cortisol levels of pigs housed in AHS or CONV measured right before (−15 min) and during a 21 h-isolation challenge. H, housing effect; T, time effect; H × T, housing × time interaction; ***P < 0.001. In case of interaction effects, within-timepoint differences between housing systems are indicated by*.

Cortisol levels in saliva (Figure 3) were affected by housing (*p* = 0.00018), time (*p* < 0.0001), and the interaction housing × time (*p* = 0.00095). In CONV pigs, cortisol concentrations decreased from baseline to +3 h, after which levels remained constant. In AHS pigs, a peak was seen at +1 h. At baseline, CONV pigs had higher levels (3.8 ± 0.54 ng/ml) compared to AHS pigs (1.3 ± 0.19 ng/ml). As a result, the estimated area under the curve related to cortisol concentration tended to be affected by the housing (*p* = 0.083) with a larger area for CONV individuals (182 ± 14 arb. unit) compared to AHS pigs (148 ± 14 arb. unit).

**Figure 4:**
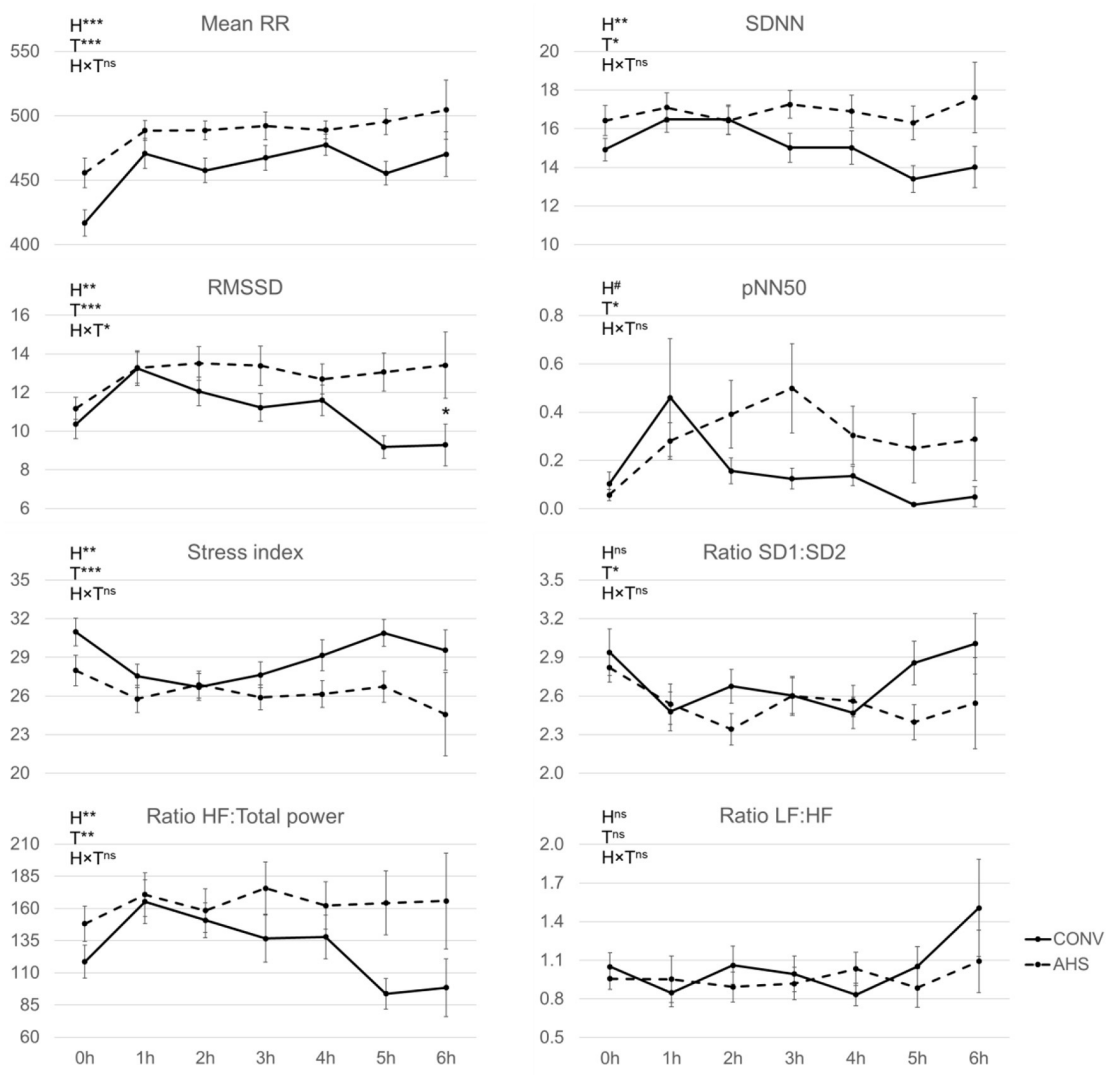
Heart rate measurements of pigs housed in AHS or CONV measured during first 6 h of an isolation challenge. H, housing effect; T, time effect; H × T, housing × time interaction; ***P < 0.001, **P < 0.01, ^#^*P* < 0.05, ^ns^not significant. In case of interaction effects, within-timepoint differences between housing systems are indicated by*.

Regarding the heart rate traits (Figure 4), RR increased from the start of the challenge to the 1st h and thereafter remained constant (time effect, *p* < 0.0001). AHS pigs showed a higher (489 ± 6.6) RR mean in ms (*p* = 0.00041), i.e., a lower heart rate (122.7 ± 1.7 bpm vs. 131.0 ± 1.9 bpm), than CONV pigs (RR mean 458 ± 6.6). RMSSD was affected by time (*p* < 0.0001), housing (*p* = 0.0037), and their interaction (*p* = 0.022). In AHS pigs, RMSSD increased from the start of the challenge to the 1st h after it was constant, whereas in CONV pigs there was the same increase from the start of the challenge to the 1st h and a drop at *t* = 5 h. As a result, at *t* = 5 h, AHS pigs had higher RMSSD than CONV pigs. The stress index, which combines different indicators, was affected by time (*p* < 0.0001) and housing (*p* = 0.0012), but was unaffected by their interaction. It decreased from the start of the challenge to the 1st h and then stayed stable. CONV pigs had a higher stress index (28.8 ± 0.69) than AHS pigs (26.4 ± 0.71). The HF: Total power ratio was also affected by time (*p* = 0.0026) and housing (*p* = 0.0059), but not by their interaction. The HF: Total power ratio increased from the start of the challenge to the 1st h and then it stayed stable. AHS pigs showed higher values (163 ±10.4) than CONV pigs (132 ± 10.4). They also had higher SDNN (16.7 ± 0.42) than CONV pigs (15.1 ± 0.41), *p* = 0.0025. SDNN increased from the start of the challenge to the 1st h and then stayed stable (time effect, *p* = 0.011). A similar temporal pattern was noticed for pNN50 (*p* = 0.023) and AHS pigs (0.19 ± 0.03) tended (*p* = 0.057) to have higher values than CONV pigs (0.11 ± 0.029). The ratio SD1:SD2 was only affected by time *(p* = 0.034) with the value at the start of the challenge being higher than the one at *t* = 1, 2, and 4 h. The ratio LF:HF was unaffected by housing, time, and their interaction.

### Cognitive Test

**Figure 5:**
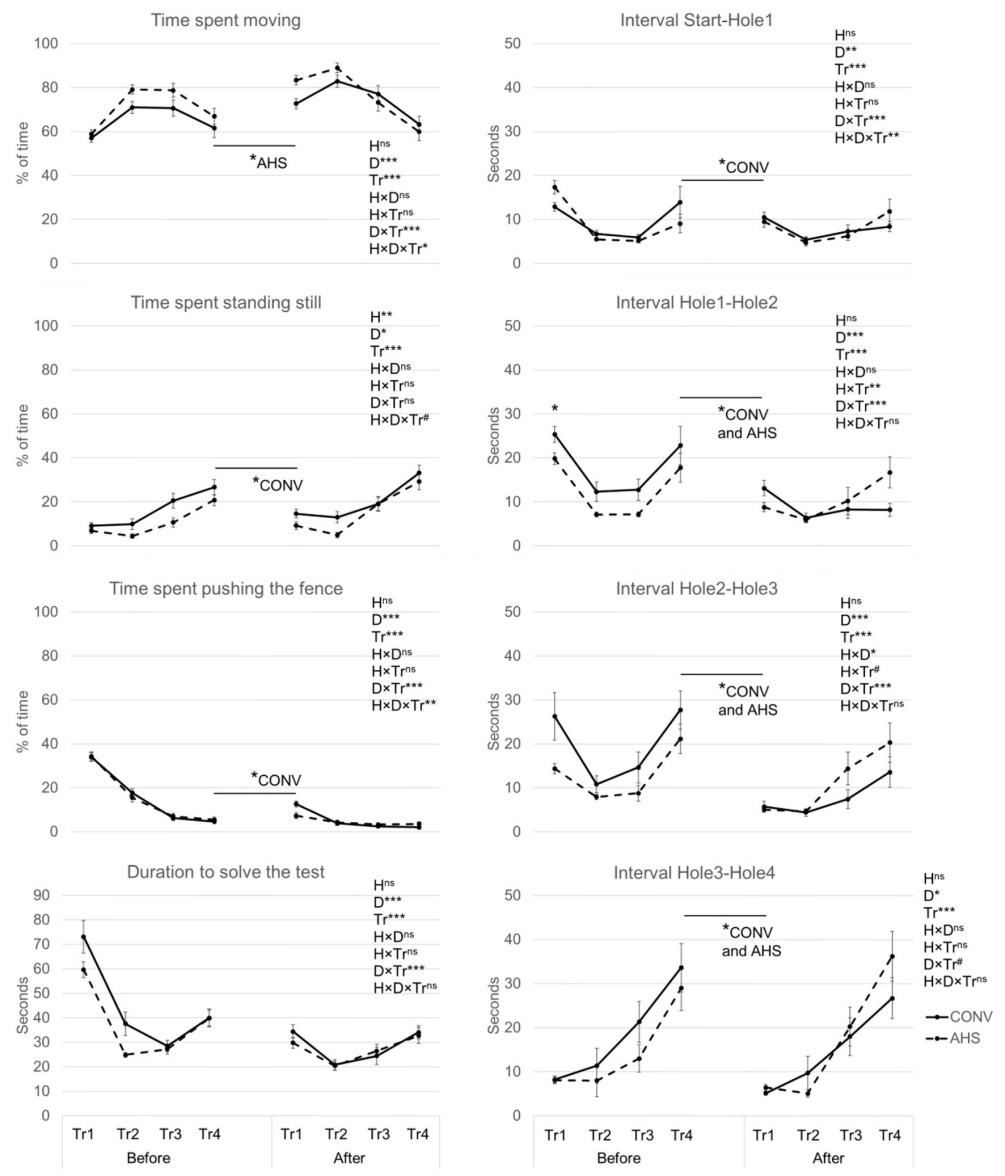
Behavioural parameters of pigs housed in AHS or CONV measured during a cognitive maze task done over four successive trials (T1, 2, 3, 4) before and after a 21-h social isolation challenge. H, housing effect; D, day effect; Tr, trial effect; H × D, housing × day interaction; H × Tr, housing × trial interaction; D × Tr, day × trial interaction; H × D × Tr, housing × day × trial interaction; ***P < 0.001, **P <0.01, *P < 0.05, ^#^*P* < 0.1, ^ns^not significant. In case of interaction effects, within-timepoint differences between housing systems are indicated by*. The figures also indicate whether and for which housing system a difference between the last trial before isolation and the last trial after isolation was found.

Figure 5 represents the changes over time of the main parameters measured during the cognitive test. The percentage of time spent moving tended to be affected by housing (*p* = 0.090), was affected by the day (*p* = 0.00052), the trial (*p* < 0.0001), the day × trial (*p* < 0.0001), and the housing × day × trial interaction (*p* = 0.038). Before isolation, both CONV and AHS pigs spent more time moving in trials 2 and 3 compared to trials 1 and 4, without a housing effect. AHS pigs moved more during the first trial after isolation compared to the last trial before isolation (*p* = 0.025) while this difference was not significant for CONV pigs. When comparing the time spent moving in trial 1 between before and after isolation, AHS pigs demonstrated a larger increase in this first trial over days than CONV pigs. After isolation, both CONV and AHS pigs moved less in trial 4 compared to trials 2 and 3. For AHS pigs, time spent moving in trial 4 was also lower than that in trial 1.

The percentage of time spent standing was affected by housing (*p* = 0.0023), by day (*p* = 0.011), and by trial (*p* < 0.0001). CONV pigs spent more time standing still (18.3 ± 1.5%) compared to AHS pigs (13.1 ± 1.5%). Pigs stood more during the cognitive test on the day after isolation (17.8 ± 1.5%) than before isolation (13.6 ± 1.5%). Trials 1 (10.0 ± 1.6%) and 2 (8.1 ± 1.6%) showed lower values compared to trial 3 (17.3 ± 1.6%), itself lower than trial 4 (27.5 ± 1.6%).

The percentage of time spent pushing the fence was affected by day (*p* < 0.0001), trial (*p* < 0.0001), and the interaction day × trial (*p* < 0.0001). Before isolation, time spent pushing the fence decreased from trial 1 (34.2 ± 1.3%) to trial 2 (16.6 ± 1.3%) till trial 3 (6.6 ± 1.3%) and then it stayed stable for trial 4 (5.1 ± 1.3%). After isolation, the decrease was only observed from trial 1 (10.0 ± 1.3%) to trial 2 (4.1 ± 1.3%) and then it stayed stable for trial 3 (3.0 ± 1.3%) and trial 4 (2.9 ± 1.3%). Time spent pushing the fence was much lower during trial 1 after the isolation compared to trial 1 before isolation, and the same holds for trial 2.

Considering the crossing of the entire maze, the duration to solve the test, i.e., reaching Hole 4 was affected by day (*p* < 0.0001), trial (*p* < 0.0001), and their interaction (*p* < 0.0001). There was no housing effect. Both before and after isolation, in trials 1 and 4 it took the pigs longer to cross the maze than in trials 2 and 3. Before isolation, the time to cross the maze was also longer in trial 1 than in trial 4, while after isolation trial 3 this was longer than in trial 2. Both in trials 1 and 2 pigs took longer to cross the maze before compared to after isolation.

The interval start-Hole1 was affected by the day (*p* = 0.0054), trial (*p* < 0.0001), and their interaction (*p* < 0.0001). Both before and after isolation, trial 1 took longer than trials 2, 3, and 4. After isolation only, trial 4 was longer than trial 2. For both trials 1 and 2, pigs were faster after isolation than before. CONV pigs needed less time (*p* = 0.0035) on the first trial after the isolation period to reach Hole1 compared to the last trial before the isolation.

The interval Hole1-Hole2 was affected by day (*p* < 0.0001), trial (*p* < 0.0001), the day × trial (*p* < 0.0001), and housing × trial interaction (*p* = 0.0013). Before and after isolation, trial 1 took longer than the other trials and there was an increase from trial 3 to trial 4. After isolation only, trial 2 was also shorter than trial 4. No matter the trial, the time to go from Hole1 to Hole2 before isolation was always higher than that after isolation. For AHS pigs only, after isolation, trial 4 showed a new rise and was significantly higher than trials 2 and 3. During trial 1, over both days, CONV pigs showed higher values than AHS pigs.

The interval Hole2-Hole3 was affected by day (*p* < 0.0001), trial (*p* < 0.0001), their interaction day × trial (*p* < 0.0001), the interaction housing × day (*p* = 0.010) without significant pairwise differences and tended to be affected by the interaction housing × trial (*p* = 0.085). Before isolation, the interval Hole2-Hole3 was longer in trials 1 and 4 than in trials 2 and 3. After isolation, only trial 2 was shorter than trial 4. No matter the trials, the interval Hole2-Hole 3 was always longer before isolation than after isolation. Both CONV and AHS pigs showed higher durations in trials 1 and 4 compared to trials 2 and 3. They also demonstrated longer intervals Hole2-Hole3 before than after isolation.

The interval Hole3-Hole4 was affected by the day (*p* = 0.036) and the trial (*p* < 0.0001) and tended to be affected by their interaction (*p* = 0.062). Before isolation, trial 1 took longer than trial 2, and trial 4 took longer than trials 2 and 3. After isolation, trial 4 was longer than all the other trials. Overall, the interval was higher before than after isolation.

Sex had no effect on any of the variables measured in the social isolation challenge or maze test.

## Discussion

In this study, resilience to social isolation was estimated by subjecting pigs to a 21 h-isolation challenge. Their cognitive resilience was assessed through measurements done during a maze task realised right after the isolation period in comparison to a neutral period before the isolation challenge. Social isolation is well-known to be stressful for pigs, which are social animals (31, 38, 48–51). Due to favourable early life experiences promoting socialisation, a more gradual and prolonged weaning transition, environmental enrichment, and extra space, which support the expression of species-specific behaviours and stimulate cognitive development, pigs from the alternative housing system were expected to develop a higher behavioural flexibility and show enhanced resilience to stressful events compared to pigs from conventional housing (52–54). This expected improved resilience would then be demonstrated in a lower expression of indicators reflecting poor welfare and tear and wear over the isolation challenge and in uncompromised cognitive abilities to solve a spatial task post-challenge.

### Response to Social Isolation

The isolation initially led to a low percentage of time spent lying during the first 15 min, which is likely related to the handling manipulations and to the exploration of the new environment by the animals. After this period, AHS pigs presented a compensatory period of resting time and stayed at commonly reported values afterwards (55–57). CONV pigs had a new disrupted period with high activity 2.5 h after the start of isolation. Although it is hard to draw conclusions about the impact of housing on the isolation challenge based on these behavioural differences, the heart rate variables showed clear housing effects. When looking at the heart rate variables measured during the isolation period, AHS pigs showed higher interbeat intervals which reflects a lower heart rate (43). Lower heart rate for the alternative housing group might either be due to a lower stress level in this specific situation, which would indicate higher resilience, or could be related to an improved cardiovascular fitness due to extra space, and therefore more activity, in their home pens (58). AHS presented higher SDNN and pNN50 which shows a higher heart rate variability related to a decrease of sympathetic tone. Those indicators may reveal a weaker sympathetic-adrenal medullary activity in response to the challenge, which could demonstrate less impact of the stressor and better welfare (43, 59). The higher RMSSD of AHS pigs at 6 h during the isolation challenge, which indicates higher integrity of vagus nerve-mediated autonomic control of the heart, and their higher HF: Total power ratio, which reveals a higher vagal activity (43, 59), is also in favour of lower stress response for those individuals. Animals with a high vagal tone are potentially less sensitive to stressful events, having greater mental, motor, and social abilities (43). The higher vagal tone in AHS animals would be in line with the expected beneficial effects provided by the alternative housing system that promotes early socialisation and species-specific behaviours for a general better welfare of the individuals.

Regarding the hormonal response during the isolation period, the pigs did not show a peak in saliva cortisol levels as compared with baseline values. The lack of a peak in response to isolation stress might be due to the sampling times selected in the current experience. In previous studies, pigs demonstrated a sharp increase in salivary cortisol between 15 and 45 min after the start of isolation (31, 39). In our study, we measured cortisol 15 min before and only 1 h after the start of the isolation challenge. Thus, it is likely that because of this sampling schedule, the peak in cortisol has been missed. During the 21 h-challenge itself, alternative housing conditions did not seem to confer any advantages in terms of stress when looking at cortisol. As previously observed in the study of Ruis et al. (31), 3 h after the start of isolation, concentrations of salivary cortisol were back to baseline levels.

At baseline, CONV pigs showed a higher cortisol concentration in saliva. They also had larger tear staining areas before the isolation compared to AHS pigs, which is an indicator of chronic stress (60). Both indicators may reflect an overall higher chronic stress level for CONV individuals, probably due to suboptimal environmental enrichment either before weaning or after and/or due to management practises from birth till 9 weeks of age. Our results are in line with other studies in which also higher basal cortisol levels for barren pigs were found in comparison to enriched ones (61, 62). On the contrary, in older pigs (from 14 weeks of age onwards), higher basal cortisol concentrations were reported for pigs housed in enriched environments compared to poor environments (22, 63). The authors suggested that the difference between the two groups might be related to the modulation of the circadian rhythm of barren pigs (flattening of the curve) due to chronic stress (22, 63, 64).

Taken together, the behavioural response and the heart rate parameters seem to indicate that AHS pigs showed fewer signs of stress in social isolation and dealt better with the challenge they were exposed to compared with CONV pigs. They also had fewer signs of chronic stress, considering the results from the tear staining and the cortisol levels, which is also in favour of an enhanced resilience for the animals kept in the alternative system.

### Cognitive Task

Before isolation, the pigs seemed to improve their performance over the first three trials. Between the first and second trials, they decreased their time spent pushing the fence, which reflects mistakes, to the benefit of more time spent moving. Consequently, the duration to reach the three first holes decreased over trials. The fourth and last trial before isolation presented, however, a different pattern with an increased duration to solve the task, less time spent moving and more time spent standing still, while not making the mistake to push the fence. The most plausible explanation is a decrease of motivation to solve the task and to reach companion pigs on the other side of the maze in favour of more time spent exploring the arena, confirmed by a longer latency to reach Hole 1 in trial 4. Pigs appeared to be less motivated by the social reward and it seemed that the relatively small length of the maze enabled them to be close enough to pen mates to feel safe and supported by their presence. This was highlighted by the length of the interval between Hole 3 and Hole 4 which was steadily increasing over trials. After crossing Hole 3, most pigs got closer to the doorstep of Hole 4 and instead of crossing it, started to chew on the wood while being really close to their pen mates. Considering the device used in this study, it is worthwhile to consider the three first holes only to evaluate the cognitive performance of the individuals, even if in our study it did not affect the results.

The housing conditions had no impact on the cognitive performance of the pigs in the maze before the isolation challenge although AHS pigs spent less time standing still, whereas these pigs were expected to perform better. Indeed, environmental enrichment may improve learning ability through the promotion of hippocampus neurogenesis and dendritic branching, which are related to memory (65–69). In line with this, Van der Beek et al. (70) found higher neuronal activity in the hippocampus of pigs housed in enriched pens compared to those in barren pens. Additionally, other studies have shown that pigs from enriched pens were able to learn a route through a maze faster (71), had improved working memory (21), better spatial memory performances (23), and made fewer mistakes in a long-term memory study than pigs from a conventional environment (63). However, other studies also found no differences between pigs from enriched or barren environments on the ability to solve a complex spatial memory task (71, 72). The inconsistency of results regarding the general effect of enrichment on cognitive skills might be related to the memory tests themselves or to differences in enrichment: period, duration, or materials. The relatively simple test in the current study may not have been challenging enough to capture subtly differences in cognitive abilities or to challenge the pigs (28, 72–74). The balance between test complexity and feasibility is difficult to find when conducting a spontaneous cognitive task: if the test is too difficult to learn and to solve, none of the individuals will succeed; if the test is too easy to solve, it will not challenge the cognitive abilities enough and all will succeed. Alternatively, potential housing effects on motivation to reach the pen mates may have obscured differences in cognition. However, the similar time to reach Hole 1 from the start box does not suggest a difference in motivation.

After the isolation challenge, similar patterns were observed in terms of behaviours, with pigs moving less in trials 3 and 4 in favour of more standing, which likely reflected the exploration of the environment. The pigs hardly pushed the fence after isolation anymore, indicating that the principle of the task was acquired. The start on the opposite side after isolation created a new route to solve the task that could have caused more mistakes, which would be partly revealed by the time spent pushing the fence. It seemed, however, that the pigs mobilised a long-term-like memory after isolation memorising the presence of holes to reach their companions. This was confirmed by their high time spent moving in trial 1 after isolation compared to before isolation, showing that the pigs did know what was expected from them. It was also reflected in the shorter durations to get from Hole 2 to 3 and from Hole 3 to 4 in the first trial after isolation, as compared with the last trial before isolation. Those improved performances right after the isolation may also be related to a stronger social motivation after the 21 h-separation to reach the pen mates at the end of the maze, or even to beneficial effects of the acute stress caused by isolation on neuroplastic mechanisms requested for cognitive performance, as suggested in rats (75) and humans studies (76–78). Some studies looked specifically at the cognitive performances of pigs around isolation: Mendl et al. (27) did find impaired spatial memory after isolation while Van der Staay et al. (34) did not find any effect on working memory.

In our study, the alternative housing conditions may have conferred a slight advantage to the AHS pigs in terms of cognitive performance after social isolation. Indeed, CONV pigs showed an increase in time spent pushing the fence in the first trial after isolation as compared with the last trial before isolation, while AHS pigs did not. CONV pigs also had a longer duration to get from the first to the second hole in trial 1 after social isolation. This may indicate a better long-term-like memory for the enriched group. This would be in line with the study of de Jong et al. (63) in which an impaired long-term memory in a maze test for barren-housed compared to enriched-housed pigs was found, while the learning abilities were not impacted.

One goal of this study was to estimate the cognitive resilience of the pigs and to determine if the alternative housing system presented beneficial effects. AHS pigs were expected to perform better, as they were expected to be more resilient to challenges and environmental enrichment was supposed to stimulate their cognitive development. Cognitive resilience can be defined as the ability to overcome the negative effects of stressors on performance or cognitive functions (30). Stress might affect the cognitive skills of an individual by impairing the specific cognitive performance. Changes in hormone concentrations are responsible for attention shifts and a decrease in decision speed (28, 79). The negative stressor selected in the current paper was 21 h-social isolation where the pigs could still hear and smell other pigs but could not see or touch them. Different isolation procedures have been used in studies on pigs. Depending on the study, pigs could or could not benefit from social support (34, 80, 81) by hearing and smelling each other, and had more or less contact with humans due to handling procedures. In the present experiment, because of saliva sampling and heart rate equipment, the pigs were handled a lot which might have lessened the impact of the isolation (53, 82, 83). The AHS and CONV pigs showed a difference in stress response to the isolation challenge before the cognitive task started. Therefore, we cannot disentangle whether the slightly better cognitive performance of AHS pigs was due to a better resilience to the isolation stressor, resulting in a lower stress load on cognition, or to an actual improved cognitive resilience, or both. Overall, we do not know whether the social isolation period applied in the current paper worsened the cognitive performance of the pigs, as the cognitive performance over time in this task was not assessed in unchallenged pigs. Indeed, both housing groups showed strong improvements regarding the duration of the intervals between holes when compared before and after isolation, making a clear conclusion on cognitive resilience difficult.

## Conclusion

The alternative housing system seemed to confer a benefit for the pigs during social isolation, which spent more time lying, and had a lower heart rate, a weaker sympathetic-adrenal medullary activity, and a higher vagal tone; all indicators being in favour of a lower impact of the stressors on the enriched pigs. The lower cortisol baseline level and tear staining area in the enriched pigs is also in line with an overall lower stress level. Hence, providing social and environmental enrichments appears to be a useful tool to improve welfare in pig husbandry. However, the housing conditions had no impact on the cognitive performance of the pigs tested before isolation. The alternative housing conditions may have conferred a slight advantage to the pigs, that seemed to have a better long-term memory-like performance. The social isolation challenge did not negatively affect the cognitive performances of the individuals, therefore the potential beneficial effects of enrichment on cognitive resilience remain unclear.

## Data Availability Statement

The raw data supporting the conclusions of this article will be made available by the authors, without undue reservation.

## Ethics Statement

The animal study was reviewed and approved by the Animal Care and Use Committee of Wageningen University (AVD1040020186245).

## Author Contributions

SP, LZ, TR, and JB designed the animal experiment. BK and EK were involved in designing the experiment. SP and LZ conducted the animal experiment. SP did the lab work, data analysis, and wrote the manuscript. All authors were involved in manuscript writing and read and approved the final manuscript.

## Funding

This study is part of the research project SmartResilience: towards a sustainable, future-oriented pig production system that supports and predicts resilience in pigs, with project number ALWGR.2017.007. The project was financed by the Netherlands Organisation for Scientific Research (NWO), and Topigs Norsvin.

## Conflict of Interest

EK was employed by Topigs Norsvin Research Center. The remaining authors declare that the research was conducted in the absence of any commercial or financial relationships that could be construed as a potential conflict of interest.

## Publisher's Note

All claims expressed in this article are solely those of the authors and do not necessarily represent those of their affiliated organizations, or those of the publisher, the editors and the reviewers. Any product that may be evaluated in this article, or claim that may be made by its manufacturer, is not guaranteed or endorsed by the publisher.
